# CIDeR: multifactorial interaction networks in human diseases

**DOI:** 10.1186/gb-2012-13-7-r62

**Published:** 2012-07-18

**Authors:** Martin Lechner, Veit Höhn, Barbara Brauner, Irmtraud Dunger, Gisela Fobo, Goar Frishman, Corinna Montrone, Gabi Kastenmüller, Brigitte Waegele, Andreas Ruepp

**Affiliations:** 1 Institute for Bioinformatics and Systems Biology (MIPS), Helmholtz Center Munich - German Research Center for Environmental Health (GmbH), Ingolstädter Landstraße 1, D-85764 Neuherberg, Germany; 2 Department of Genome-oriented Bioinformatics, Life and Food Science Center Weihenstephan, Technische Universität München, Alte Akademie 1, 85354 Freising, Germany

## Abstract

The pathobiology of common diseases is influenced by heterogeneous factors interacting in complex networks. CIDeR http://mips.helmholtz-muenchen.de/cider/ is a publicly available, manually curated, integrative database of metabolic and neurological disorders. The resource provides structured information on 18,813 experimentally validated interactions between molecules, bioprocesses and environmental factors extracted from the scientific literature. Systematic annotation and interactive graphical representation of disease networks make CIDeR a versatile knowledge base for biologists, analysis of large-scale data and systems biology approaches.

## Rationale

Complex diseases such as diabetes and Alzheimer's disease are increasingly important causes of illness and mortality burden, and they are leading drivers of healthcare costs, constituting an important burden for societies in both developed and developing countries around the world. It is projected that by 2025 there will be 380 million people with type 2 diabetes world-wide [1]. Thus, elucidating the genetic and non-genetic determinants of complex human diseases represents one of the principal challenges of biomedical research. In the course of the last decades advances in our understanding of pathobiological processes in complex diseases were mainly driven by individual experiments dedicated to particular aspects of the individual diseases. It could be shown that a disease phenotype is the result of pathobiological processes that interact in complex networks. Members in these networks consist of various types of interacting biomolecules involved in bioprocesses influenced by genetic and environmental factors. Analyses of the multiple types of interconnections between these factors are performed in systems biology approaches and have also been coined 'network medicine' [2].

In recent years, technical advances in high-throughput SNP analyses laid the foundation for genome-wide association studies. Despite the success of genome-wide association studies in identifying loci associated with common diseases, a substantial proportion of the causality remains unexplained [3]. In a recent study, a network-based approach has been used successfully to identify interconnections between candidate genes that were identified in a deep sequencing approach for recessive cognitive disorders [4].

**Figure 1:**
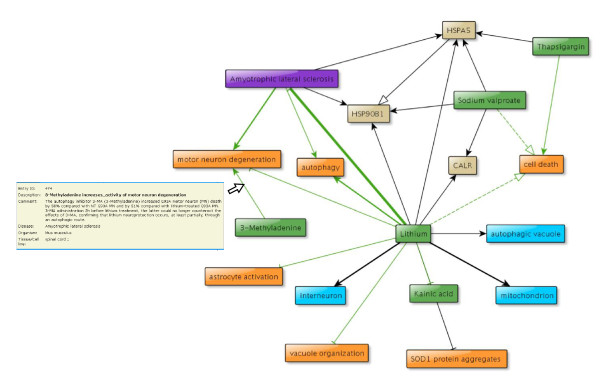
**Graphical presentation of a lithium interaction network in CIDeR**. The graph shows the interaction network of lithium in bipolar disorder and amyotrophic lateral sclerosis together with functional interactions between proteins (beige), chemical compounds (green), cell types (light blue) and biological processes (orange). To the left, a pop-up window is open that shows detailed curated information related to the interaction between '3-Methyladenine' and 'motor neuron degeneration'. CALR, calreticulin; SOD, superoxide dismutade.

However, there is a lack of disease-related resources that allow analysis of disease-associated factors integrated in a network structure. Available disease diagrams as provided by the Kyoto Encyclopedia of Genes and Genomes (KEGG) [5] and using the CellDesigner software [6] allow obtaining a broad outline about basic disease concepts but are not designed as comprehensive resources. Here, we present CIDeR, a database with manually curated information from neurological and metabolic diseases. CIDeR has been developed to facilitate systems-level analyses for providing better insight into the complex networks of pathways and interactions that govern pathobiological processes in human diseases. Multiple search options and interactive graphical presentation of networks (Figure 1) enable inspection of the manifold interrelations between heterogeneous disease factors that are required for the understanding of disease etiology.

## Manual curation of interactions in disease processes

**Figure 2:**
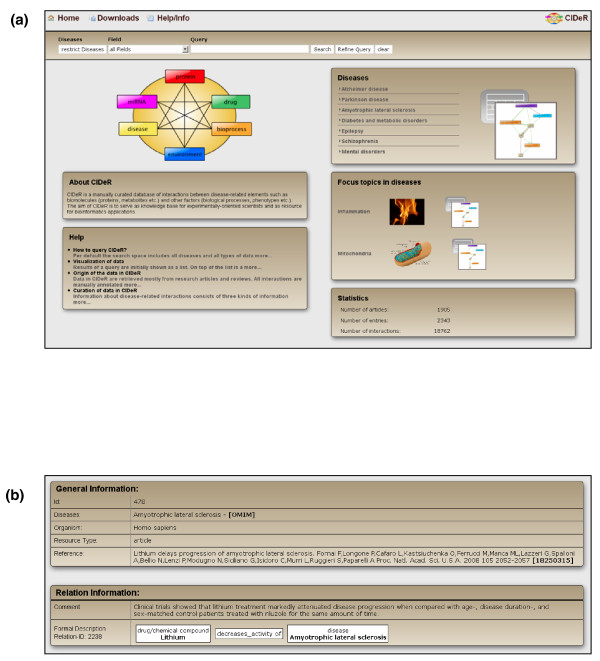
**The CIDeR home page and curation of an interaction**. **(a) **The CIDeR home page contains statistics, search options as well as links to focus topics and disease pages. **(b) **A manually curated interaction containing general information, textual information (comment), and structured information (formal description).

CIDeR covers disease-related interactions from neurodegenerative diseases (Alzheimer's disease, Parkinson's disease, amyotrophic lateral sclerosis (ALS)), mental disorders (schizophrenia, depression) as well as the metabolic diseases (type 2 diabetes). Most of our current knowledge about disease processes has been generated by numerous individual experiments shedding light on certain aspects of a disease. The results of these studies describe interactions between entities such as proteins but also, for example, the influence of an external stimulus on protein expression or the influence of cellular compounds on bioprocesses. An interaction is defined as the relation between two objects (proteins, chemical compounds, and so on) that affect each other or change each other (for example, by activation, modification or binding). The vast amount of experimental findings is hidden in the textual information of the biomedical literature. Existing thesauri for proteins and chemical compounds support searches in resources like PubMed [7] or using text-mining approaches [8]. However, heterogeneous and ambiguous descriptions in areas like cellular processes or phenotypes hamper the detection and processing of published information [8]. To enable information extraction from the biomedical publications comprehensively and with high-quality the complete database content of CIDeR has been generated by experienced biocurators who read and manually curate the complete articles from peer-reviewed scientific literature. For the selection of appropriate articles we make use of automated text mining [9], searches in PubMed and information from referenced literature. The approach of annotating diseases in CIDeR is to collect information of disease processes that is required to describe the current knowledge of the pathobiology of common diseases. The process-oriented strategy allows assembling the interconnections between various disease factors into integrated networks. Using manual curation enables translation of complicated topics into established vocabularies to provide information for further analysis. It is, for example, not apparent that the 'Morris water maze task' is used to test spatial learning and memory of mammalian model organisms [10]. Manual curation allows annotating respective results by using the Gene Ontology terms 'GO:0008542: visual learning' and 'GO:0007613: memory'. Furthermore, detailed manual curation permitted us to richly annotate the interactions and to place them in their relevant context. This contextual annotation includes details like the species used in the experiments, gender, use of model organisms, supporting publication, cell type, cell-line, tissue, and other aspects. As of May 2012, we have reviewed 1,912 publications and curated 18,813 disease-relevant interactions (Figure 2).

## Data structure of disease interactions

For transformation of the biomedical information into a data structure suitable for bioinformatics applications as well as for wet lab scientists, interactions in CIDeR consist of three parts (Figure 2): structured information, textual comment, and general information.

### Structured information

The core element of disease annotation in CIDeR is the structured information describing the interaction between two elements as a formal subject-predicate-object structure (for example, protein A increases_activity_of protein B). A similar structure is used in resources such as Drugbank [11] or in the process diagram editor CellDesigner [6]. Subjects and objects can be molecules such as nucleic acids, proteins or chemical compounds and other elements like cellular processes, phenotypes or environmental factors. In order to provide the content of CIDeR in a standardized format, we use names and identifiers from established resources like EntrezGene [12], KEGG [5], CORUM [13] or Online Mendelian Inheritance in Man (OMIM) [14] for annotation. The structured information is required for analysis of the interactions via bioinformatics methods. However, a caveat of this type of data is that it is restricted to the basic information of an experiment and thus does not include important details of the experimental approach or results.

### Textual comment

This kind of information is found in the textual information of interactions in CIDeR. It is especially useful for experimentally working scientists to know at what concentrations a drug has been applied in an experiment and it is of general interest to know to what extent the expression of a protein has been increased. The textual information provides an integrated view aiming for combining information from different sources in a comprehensive form.

### General information

The general information includes literature references, the respective disease, and information describing the experimental setup. This information is required to be aware whether a finding holds true for all organisms or only for individuals with a specific genetic or experimental disposition. For example, there is growing evidence that the gender has an important role in disease processes. For type 2 diabetes it was shown that Gly482Ser polymorphism of PGC-1alpha (encoded by *PPARGC1A*) was associated with a lower plasma adiponectin level in type 2 diabetic men but not in type 2 diabetic women [15]. In neurological diseases, gender-specific disease processes were also found. XBP1 deficiency in female mutant SOD1 transgenic mice resulted in a highly significant increase in life span of 22 days [16].

Interaction data from CIDeR can be downloaded as flat files or in Systems Biology Markup Language (SBML), a free and open interchange XML format for computer models of biological processes [17]. Files in the SBML format can also be loaded with network analysis tools such as Cytoscape [18]. Besides downloading of the complete database, CIDeR also enables downloading of interactions from search results. Graphical outputs can be downloaded in JPEG format or GraphML format. The latter can be opened and edited via the graph editor yED (yWorks GmbH, Tübingen, Germany).

## Search options and visualization of interaction networks

A flexible web-based interface [19] allows both simple and more advanced searching of CIDeR. The interface has been developed in close collaboration with experimental biologists to ensure that the interface is intuitive and easy to use for the end user. In order to allow defining a precise search space, CIDeR offers various search options. By default all disease interactions are queried, but the user has the option to define the search for only a set of diseases. This is especially useful when processes from different diseases are interconnected. Evidence was found that related disease-associated cellular processes are affected in Alzheimer's disease and type 2 diabetes. In the Rotterdam study, it was shown that diabetes mellitus almost doubled the relative risk of dementia and Alzheimer's disease [20].

Complex search and analysis options permit more powerful systems-level views of the disease relevant processes. The options include, among others, the ability to search for, for example, 'Gene/Protein/Complex', 'Biological process', 'Tissue/cell line' or 'Chemical compound'. To make this process intuitive, all of the possibilities are listed in a 'dropdown box'. Molecule searches allow searching for a particular gene or chemical compound of interest, either by name or via identifiers from external databases. In addition, CIDeR stores lists of all synonyms from Entrez Gene [12] and KEGG [5]. Gene or chemical compound searches of CIDeR automatically search all possible alternative names for the query rather than restricting searches to particular names or symbols from a specific database. If an initial search has been finished, it can be refined with a second search by using the 'refine query' option. Results of the second search term are associated with the first search term by one of the three operators 'AND', 'OR' and 'NOT'. The search results appear as a table that links to the curated content of the interactions or to a graph tool that dynamically generates a graph from the search results (Figure 1; Figures s1, s2, s3, and s4 in Additional file 1).

All disease interactions can be interactively investigated using the yFiles framework (yWorks GmbH) with an AJAX extension that automatically generates more biologically intuitive pathway-like layouts of a network. Within the graph, interactions are shown as color-coded nodes (in graphs, nodes are objects such as a protein, chemical compound or bioprocesses) that are linked via defined relations (edges). The graph tool enables generation of an optimized layout of the graph elements and also allows implementation of interactive tools. While moving the mouse cursor over edges, pop-up windows with respective interaction information appear (Figure 1). A click on the edge opens a window with the complete annotated information about this interaction. Another useful function is the extension of the graph for an element of interest. If the user wants in addition to show all interactions that are linked to a certain node, he just needs to double-click on the element.

In addition to the ability to generate user-defined graphs, CIDeR provides predefined graphs (Focus topics) on the homepage displaying the role of inflammation and mitochondria for the annotated diseases and concomitantly offering an entry point in order to explore the structure of disease networks in CIDeR (Figure 2). Here, additional predefined graphs for disease-relevant subjects like the interconnection between Parkinson's disease and cancer are shown. In addition, these pages offer statistics about proteins, chemical compounds, and biological processes that are hyperlinked to the respective content. The generation of novel 'Focus topics' is an ongoing activity that sheds light on current developments in disease research.

## Comparison of CIDeR with existing resources

An early example of an effort to display biological processes in graphical networks is the biochemical pathways from Gerhard Michal [21]. Despite several resources such as Reactome [22] and PharmGKB [23] that present biochemical pathways in network structure, there are only few approaches providing disease information in a network structure. Two notable, publicly available examples are KEGG pathways [5] and process diagrams constructed with CellDesigner [24]. The conceptual approach of CIDeR offers advantages compared to disease information from KEGG and CellDesigner. First, CIDeR is an extensive, manually curated resource that mostly provides direct experimental evidence for the interactions and also associated context information. Disease information in KEGG is also collected by manual curation but is mainly based on review information. As a result, biomedical information in CIDeR provides results from investigations in more depth and, in addition, CIDeR presents important context information such as cell lines and gender of organisms. Second, in contrast to KEGG and CellDesigner, CIDeR offers multiple search options enabling specific areas of diseases to be focused on and the results to be presented in dynamically generated graphs. Graphical representation in KEGG and CellDesigner is static and shows the complete disease information in a single diagram. Static diagrams are useful for presentation of the scientific progress at the time of creation. However, inclusion of novel findings into complex process diagrams like in CellDesigner is hardly feasible. Third, disease information in CellDesigner and especially KEGG is limited to the basic concepts of diseases. The type 2 diabetes diagram in KEGG [5] consists of 36 interactions whereas CIDeR currently presents 6,911 interactions for the disease. The limited amount of data in KEGG is a result of the static diagrams, as these become hardly comprehensible if a large amount of details has to be shown. Fourth, CIDeR includes a wide range of disease-relevant molecules as well as other disease relevant factors such as bioprocesses and phenotypes. Disease annotation in KEGG and CellDesigner is concentrated on interactions between proteins. An extension of the KEGG dataset is available from the PROMISCOUS database [25] that includes the protein-drug interactions from Drugbank [11]. Fifth, information from CIDeR can be downloaded (see below) as a complete database but also areas of interest that are generated by searches. Download in KEGG always includes the complete dataset. Users that are interested in a particular aspect of the disease have to sort out the respective interactions manually.

A further resource providing manually curated disease interactions is the Comparative Toxicogenomics Database (CTD). CTD provides a manually curated triad of chemical-gene, chemical-disease and gene-disease relationships from the literature [26]. For these three types of interactions, CTD provides 260,300 data points covering a large variety of diseases. The conceptual difference between the two resources is that CTD stores a large number of valuable interactions whereas CIDeR adopts a disease process-oriented approach and facilitates the inspection of interconnected disease networks. Therefore, the description of the pathobiological processes for this complex disease in CIDeR requires at least 40 different types of interactions (see below). As a consequence of the process-driven approach CIDeR enables interactive visualization of disease-networks, which is not available for CTD. In addition, CIDeR provides a more in-depth annotation including contextual information as described above.

The quantitative comparison reveals that the different approaches of CIDeR, KEGG, CTD and CellDesigner result in significant differences with respect to the variety and absolute numbers of interactions (Tables s1, s2, s3, and s4 in Additional file 2). Metabolic syndrome/type 2 diabetes is a disorder that is covered by all four resources. KEGG, which covers a large spectrum of different bioprocesses and diseases, uses 36 interactions and two different interaction types (gene-gene and gene-chemical compound) in order to annotate type 2 diabetes. The CTD database provides a total of 21,791 interactions distributed on two interaction types (chemical-disease and gene-disease) for diabetes type 2. The chemical-compound gene interactions from CTD could not be included in the statistical comparison as they are not assigned to defined diseases and are, to some extent, not disease-associated at all. Annotation of metabolic syndrome in SBML format [17] with diagrams created using CellDesigner make use of eight different interaction types and 97 interactions. For annotation of diabetes/metabolic syndrome in CIDeR we have currently annotated 7,165 disease-interactions that are found in 40 different interaction types. In conclusion, the quantitative analysis of the different resources results in few interactions for the general overview given in KEGG, an extensive set of data restricted to two disease interaction types in CTD and a large number of different interaction types for the network-oriented approach in CIDeR.

A large fraction of other disease-related resources is dedicated to particular diseases or specific molecules. SZgene provides a field synopsis of genetic association studies in schizophrenia [27], dbDEPC is a database of differentially expressed proteins in human cancers [28] and MDPD is a mutation database for Parkinson's disease [29]. All these databases contain invaluable information for disease research but they do not support approaches in the fields of systems biology and network medicine.

## Applications of the CIDeR database

The aim of CIDeR is to provide a knowledge resource for experimentally working scientists as well as to present structured information for systems biology and analyses of high-throughput experiments. In-depth manual annotation of scientific literature offers wet-lab scientists a substantial structured overview about disease processes. This information is highly suitable to generate qualitative models and hypotheses to be tested experimentally. Especially the simultaneous inspection of results within a disease area such as neurological diseases overcomes the limits of intuitive but inefficient trial and error approaches. An example of the benefit that can be derived from comparing different research fields is the mood stabilizer lithium (Figure 1), a psychiatric drug that has been the standard pharmacological treatment for bipolar disorder for many years [30]. In a recent study it was found that daily doses of lithium delay disease progression in human patients affected by ALS [31]. In the same study a neuroprotective effect of lithium could be demonstrated, which is well in line with the observation that lithium has neurotrophic effects in bipolar disorder [32].

Inspection of a disease network can reveal the multiple interactors of lithium, such as glycogen synthase kinase-3 (GSK3), calreticulin or the heat shock protein HSPA5, which are proposed to mediate lithium action. If lithium delays disease progression in human patients affected by ALS according to [31] and lithium directly interacts with GSK3, calreticulin and HSP5, the expression of the genes encoding these three proteins might serve as an indicator of disease development (Figure 1).

Information about different diseases in CIDeR also allows detection of potential side effects of drugs, such as adverse reactions. HSD11B1 (11-beta hydroxysteroid dehydrogenase type 1) catalyzes the conversion of inactive cortisone to active cortisol. The discovery that increased cortisol levels are involved in the etiology of metabolic syndrome and type 2 diabetes gave rise to the development of the HSD11B1 inhibitors INCB13739 and MK-0916 [33,34]. Since polymorphisms in the *HSD11B1 *gene have been associated with increased risk for Alzheimer's disease [35] and susceptibility to incident depression [36], HSD11B1 inhibitors should specifically be tested for their effects in patients affected by Alzheimer's disease or depression (Additional file 3).

Systems biology represents a general integrative strategy for the understanding of complex biological systems [37]. Currently, approaches exist that allow dynamic modeling - for example, of T-cell receptor signaling, with 94 nodes [38]. The first step in modeling is construction of a qualitative network describing the mode of interaction between the components of a network. Inclusion of experimental data subsequently enables the design of quantitative models allowing for a parameterized prediction of observable readouts leading to a deeper understanding of perturbed molecular mechanisms in disease processes. Due to the structured annotation of the CIDeR content, the dataset is well suited to construct qualitative disease networks on a rational basis while saving time and effort for extensive literature research.

To expedite the molecular elucidation of human diseases, we will face an avalanche of data from genome sequencing and exome sequencing experiments in the years to come [39]. The challenge for the interpretation of high-throughput -omics data lies in assimilating it in the context of existing knowledge. Currently, this step is often restricted to Gene Ontology [40] enrichment analysis, allowing only very general but intuitive conclusions. As an example beyond mapping to functional categories, a recent study for autosomal-recessive intellectual disability has revealed probable disease-causing variants in 50 novel candidate genes [4]. With the help of protein-network analysis it could be shown that novel candidate genes are within functional interaction networks together with previously known intellectual disability genes. For such network-oriented approaches CIDeR can be useful as it interconnects proteins not only by protein-protein interactions but also by linking proteins via factors such as microRNAs, chemical compounds, bioprocesses, and environmental factors.

In conclusion, CIDeR provides a publicly available integrative resource for researchers in neurological and metabolic diseases as well as for the broader life science community. CIDeR has been developed to present biological facts in an intuitively displayed molecular interaction network and pathway context, which will enable scientists without a strong computational background to explore their data in a more systems-oriented manner.

## Abbreviations

ALS: amyotrophic lateral sclerosis; CTD: Comparative Toxicogenomics Database; KEGG: Kyoto Encyclopedia of Genes and Genomes; SBML: Systems Biology Markup Language; XML: Extensible Markup Language.

## Competing interests

The authors declare that they have no competing interests.

## Authors' contributions

BB, ID, GF, GMF and CM contributed to the literature curation process and participated in the study design. ML, VH, GK and BW implemented the database and the web design. AR conceived the project and wrote the manuscript. All authors read and approved the final manuscript.

## Supplementary Material

Additional file 1 **Supplementary figures**. Figure s1: Go to the CIDeR homepage: Focus topics in diseases => mitochondria. Choose 'Mental disorders' and click on 'Graph'. A user who is interested in a research area, such as mitochondria, that is prepared as focus topic has access to this information via links on the CIDeR homepage. In order to obtain more or more specific information on the topic, the user has the option to start an individual search (Figure s2). Figure s2: CIDeR query: mitochondrial respiratory chain complex V. The user starts a survey in CIDeR - for example, with a query for 'Mitochondrial respiratory chain complex V' - and clicks on 'Graphical View'. Knowledge of the precise term is not required as CIDeR makes use of an auto-complementation tool that offers all related terms in the database after a few characters have been filled in. The search for 'Mitochondrial respiratory chain complex V' results in a graph consisting of six elements (nodes). It shows that the mitochondrial respiratory chain complex V is linked to bipolar disorder as well as to two angiotensin receptors (AGTR1 and AGTR2), which are all part of the angiotensin-renin system, which regulates blood pressure but also interacts with insulin signaling and is thus part of the type 2 diabetes network. A double-click on AGTR1 (Figure s3) extends the 'Mitochondrial respiratory chain complex V' for the AGTR1 network. Figure s3: CIDeR query: Mitochondrial respiratory chain complex V and double-click on AGTR1. A double-click on AGTR1 shows relations between AGTR1 and several drugs - for instance, nebivolol, a beta-blocker. Nebivolol interacts with a number of proteins and processes, many of them involved in blood pressure regulation, but also insulin resistance. AGTR1 also shows an interaction with cortisol (Figure s4). Figure s4: CIDeR query: Mitochondrial respiratory chain complex V and double-click on AGTR1 and double-click on cortisol. A double click on cortisol leads to interactions with different diseases (Alzheimer's disease, schizophrenia, insulin resistance) as well as to inflammatory response, a bioprocess that is involved in the etiology of various diseases and is also curated in CIDeR as 'Focus topic'. The figure shows that different diseases, such as bipolar disorder and insulin resistance, can be linked via a few elements ('Mitochondrial respiratory chain complex V'-AGTR1-cortisol) and that a disease network is created that can facilitate the generation of hypotheses - for example, to investigate diseases using drugs that are not ordinarily used for them. Click here for file

Additional file 2 **Supplementary tables**. Table s1: quantitative comparison of interaction information in different resources - diabetes/metabolic syndrome. The table shows the different types of interactions (process-phenotype, environment-process, microRNA-genes, and so on) and the number of respective interactions for diabetes/metabolic syndrome. Table s2: quantitative comparison of interaction information in different resources - Alzheimer's disease. The table shows the different types of interactions (process-phenotype, environment-process, microRNA-genes, and so on) and the number of respective interactions for Alzheimer's disease. Table s3: quantitative comparison of interaction information in different resources - Parkinson's disease. The table shows the different types of interactions (process-phenotype, environment-process, microRNA-genes, and so on) and the number of respective interactions for Parkinson's disease. Table s4: quantitative comparison of interaction information in different resources - amyotrophic lateral sclerosis. The table shows the different types of interactions (process-phenotype, environment-process, microRNA-genes, and so on) and the number of respective interactions for amyotrophic lateral sclerosis. Click here for file

Additional file 3 **Potential side effects of type 2 diabetes drugs on patients affected by Alzheimer's disease or depression**. Click here for file
