# Supplementary material for: CIDeR: multifactorial interaction networks in human diseases
Source: Genome Biol. 2012 Jul 18;13(7):R62. doi: 10.1186/gb-2012-13-7-r62 (PMC3491383; doi:10.1186/gb-2012-13-7-r62)

Additional Figure s1

CIDeR homepage: **Focus topics in diseases** => **mitochondria**

Choose ‚**Mental disorders**‘ and click on ‚**Graph**‘

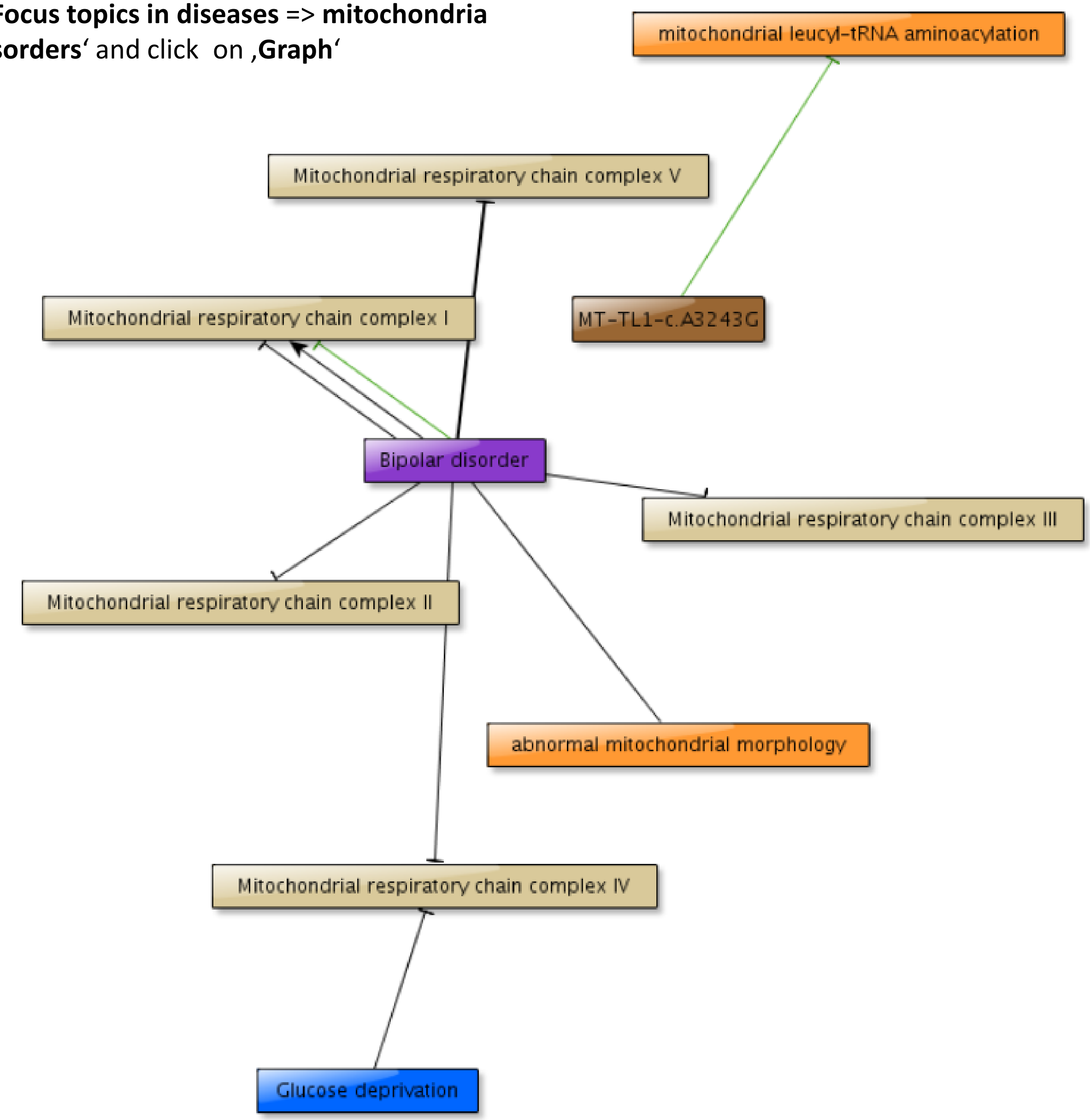

Additional Figure s2
CIDeR homepage
Query: **Mitochondrial respiratory chain complex V**

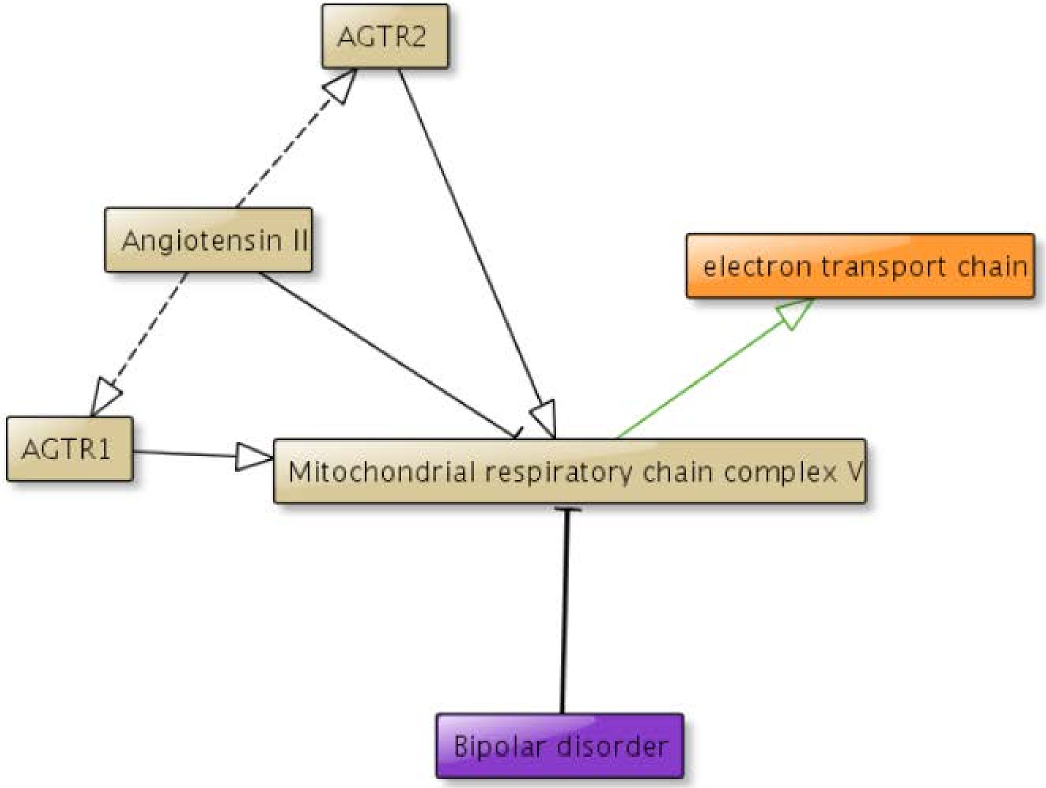

Additional Figure s3
CIDeR homepage
Query: **Mitochondrial respiratory chain complex V**
and Click on **AGTR1**

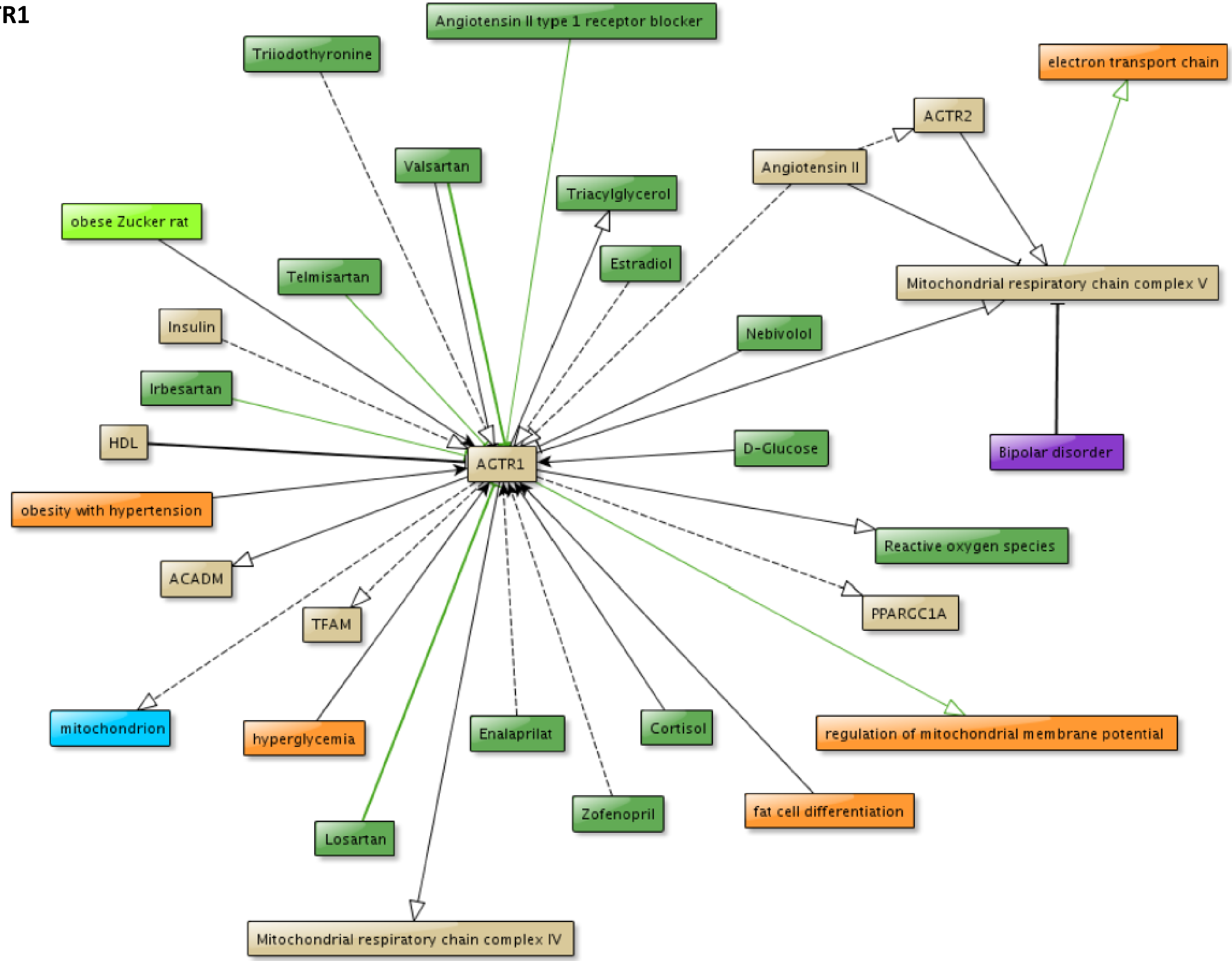

Additional Figure s4
CIDeR homepage
Query: **Mitochondrial respiratory chain complex V**
and click on **AGTR1**
and click on **Cortisol**

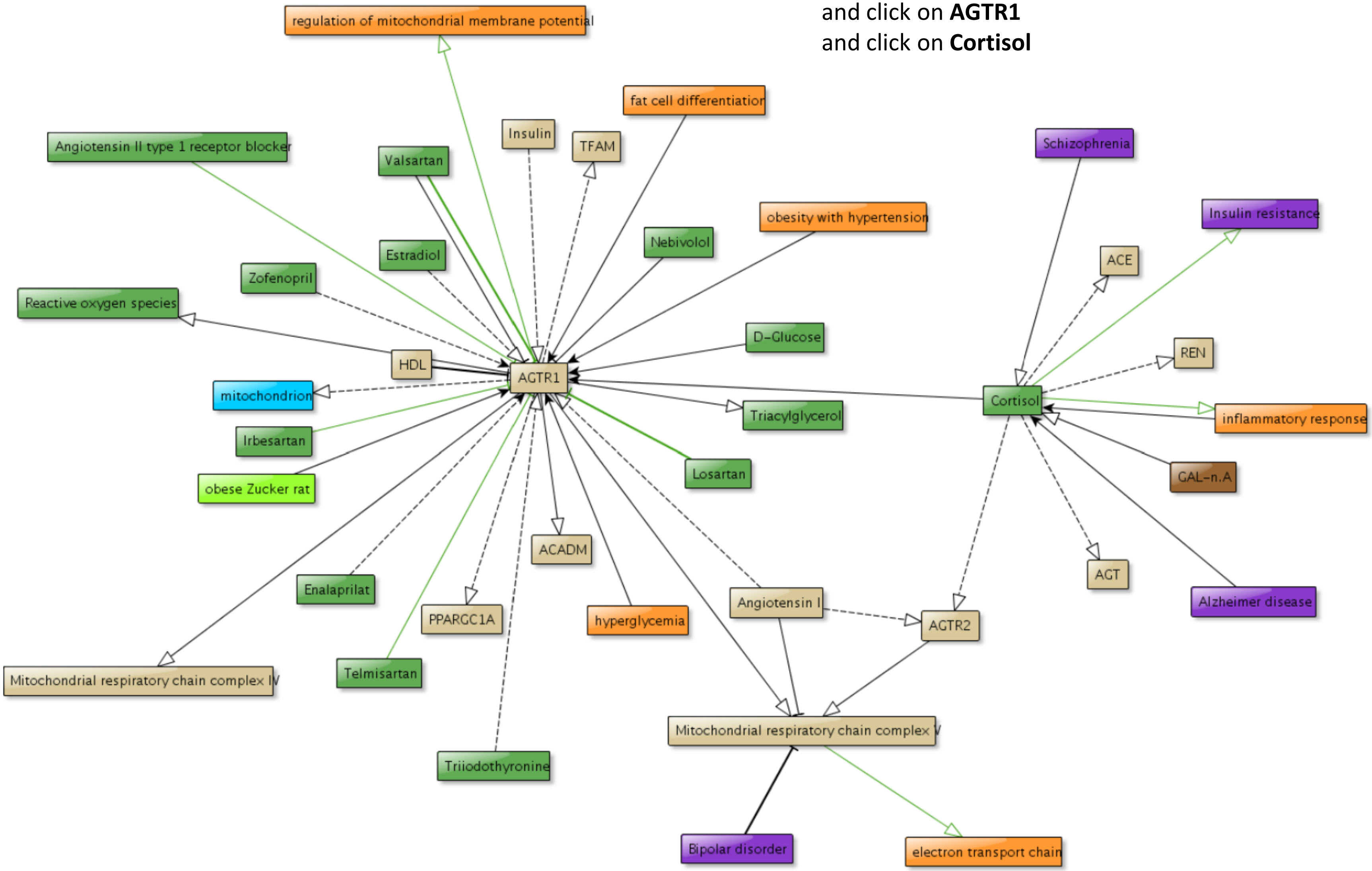

Supplement: Additional file 1 — Supplementary figures. Figure s1: Go to the CIDeR homepage: Focus topics in diseases => mitochondria. Choose 'Mental disorders' and click on 'Graph'. A user who is interested in a research area, such as mitochondria, that is prepared as focus topic has access to this information via links on the CIDeR homepage. In order to obtain more or more specific information on the topic, the user has the option to start an individual search (Figure s2). Figure s2: CIDeR query: mitochondrial respiratory chain complex V. The user starts a survey in CIDeR - for example, with a query for 'Mitochondrial respiratory chain complex V' - and clicks on 'Graphical View'. Knowledge of the precise term is not required as CIDeR makes use of an auto-complementation tool that offers all related terms in the database after a few characters have been filled in. The search for 'Mitochondrial respiratory chain complex V' results in a graph consisting of six elements (nodes). It shows that the mitochondrial respiratory chain complex V is linked to bipolar disorder as well as to two angiotensin receptors (AGTR1 and AGTR2), which are all part of the angiotensin-renin system, which regulates blood pressure but also interacts with insulin signaling and is thus part of the type 2 diabetes network. A double-click on AGTR1 (Figure s3) extends the 'Mitochondrial respiratory chain complex V' for the AGTR1 network. Figure s3: CIDeR query: Mitochondrial respiratory chain complex V and double-click on AGTR1. A double-click on AGTR1 shows relations between AGTR1 and several drugs - for instance, nebivolol, a beta-blocker. Nebivolol interacts with a number of proteins and processes, many of them involved in blood pressure regulation, but also insulin resistance. AGTR1 also shows an interaction with cortisol (Figure s4). Figure s4: CIDeR query: Mitochondrial respiratory chain complex V and double-click on AGTR1 and double-click on cortisol. A double click on cortisol leads to interactions with different diseases [file gb-2012-13-7-r62-S1.PDF]
