# Supplementary material for: CIDeR: multifactorial interaction networks in human diseases
Source: Genome Biol. 2012 Jul 18;13(7):R62. doi: 10.1186/gb-2012-13-7-r62 (PMC3491383; doi:10.1186/gb-2012-13-7-r62)
Supplement: Additional file 2 — Supplementary tables. Table s1: quantitative comparison of interaction information in different resources - diabetes/metabolic syndrome. The table shows the different types of interactions (process-phenotype, environment-process, microRNA-genes, and so on) and the number of respective interactions for diabetes/metabolic syndrome. Table s2: quantitative comparison of interaction information in different resources - Alzheimer's disease. The table shows the different types of interactions (process-phenotype, environment-process, microRNA-genes, and so on) and the number of respective interactions for Alzheimer's disease. Table s3: quantitative comparison of interaction information in different resources - Parkinson's disease. The table shows the different types of interactions (process-phenotype, environment-process, microRNA-genes, and so on) and the number of respective interactions for Parkinson's disease. Table s4: quantitative comparison of interaction information in different resources - amyotrophic lateral sclerosis. The table shows the different types of interactions (process-phenotype, environment-process, microRNA-genes, and so on) and the number of respective interactions for amyotrophic lateral sclerosis. [file gb-2012-13-7-r62-S2.PDF]

Table S1

Resource: CIDeR
Disease: Diabetes/metabolic syndrome
Interactions: 7165

	genes/proteins	tissue/cell line	process	phenotype	microRNA	environment	drug/chemical compound	disease	cellular component	organism model
genes/proteins	1725									
tissue/cell line	40	1								
process	855	7	32							
phenotype	587	0	51	21						
microRNA	1	0	1	4	0					
environment	398	7	51	22	0	1				
drug/chemical compound	1409	21	229	142	1	46	158			
disease	883	3	59	52	1	19	130	34		
cellular component	36	0	0	0	0	0	4	0	0	
organism model	100	0	10	14	0	0	1	8	1	0

Resource: CellDesigner (PMID:15561923)
Disease: Metabolic syndrome
Interactions: 97

	genes/proteins	tissue/cell line	process	phenotype	microRNA	environment	drug/chemical compound	disease	cellular component	organism model
genes/proteins	7									
tissue/cell line	0	0								
process	1	0	3							
phenotype	0	0	0	0						
microRNA	0	0	0	0	0					
environment	0	0	0	0	0	0				
drug/chemical compound	10	0	17	0	0	0	49			
disease	0	0	0	0	0	0	0	0		
cellular component	0	0	2	0	0	0	8	0	0	
organism model	0	0	0	0	0	0	0	0	0	0

Resource:	CTD
Disease:	Diabetes mellitus, type 2
Interactions:	21,791

	genes/proteins	tissue/cell line	process	phenotype	microRNA	environment	drug/chemical compound	disease	cellular component	organism model
genes/proteins	0									
tissue/cell line	0	0								
process	0	0	0							
phenotype	0	0	0	0						
microRNA	0	0	0	0	0					
environment	0	0	0	0	0	0				
drug/chemical compound	0	0	0	0	0	0	0			
disease	16609	0	0	0	0	0	5182	0		
cellular component	0	0	0	0	0	0	0	0	0	
organism model	0	0	0	0	0	0	0	0	0	0

Resource:	KEGG
Disease:	Diabetes mellitus, type 2
Interactions:	36

	genes/proteins	tissue/cell line	process	phenotype	microRNA	environment	drug/chemical compound	disease	cellular component	organism model
genes/proteins	27									
tissue/cell line	0	0								
process	0	0	0							
phenotype	0	0	0	0						
microRNA	0	0	0	0	0					
environment	0	0	0	0	0	0				
drug/chemical compound	9	0	0	0	0	0	0			
disease	0	0	0	0	0	0	0	0		
cellular component	0	0	0	0	0	0	0	0	0	
organism model	0	0	0	0	0	0	0	0	0	0

Table S2

Resource: CIDeR
Disease: Alzheimer
Interactions: 3153

	genes/proteins	tissue/cell line	process	phenotype	microRNA	environment	drug/chemical compound	disease	cellular component	organism model
genes/proteins	741									
tissue/cell line	64	3								
process	289	14	55							
phenotype	147	6	35	18						
microRNA	39	5	6	1	1					
environment	18	0	3	6	0	0				
drug/chemical compound	365	17	173	63	3	1	52			
disease	347	4	58	91	36	10	102	16		
cellular component	99	0	4	10	1	0	12	6	0	
organism model	152	1	27	29	9	0	7	6	1	0

Resource: CTD
Disease: Alzheimer
Interactions: 18,799

	genes/proteins	tissue/cell line	process	phenotype	microRNA	environment	drug/chemical compound	disease	cellular component	organism model
genes/proteins	0									
tissue/cell line	0	0								
process	0	0	0							
phenotype	0	0	0	0						
microRNA	0	0	0	0	0					
environment	0	0	0	0	0	0				
drug/chemical compound	0	0	0	0	0	0	0			
disease	9768	0	0	0	0	0	9031	0		
cellular component	0	0	0	0	0	0	0	0	0	
organism model	0	0	0	0	0	0	0	0	0	0

Resource:	KEGG
Disease:	Alzheimer
Interactions:	25

	genes/proteins	tissue/cell line	process	phenotype	microRNA	environment	drug/chemical compound	disease	cellular component	organism model
genes/proteins	21									
tissue/cell line	0	0								
process	0	0	0							
phenotype	0	0	0	0						
microRNA	0	0	0	0	0					
environment	0	0	0	0	0	0				
drug/chemical compound	4	0	0	0	0	0	0			
disease	0	0	0	0	0	0	0	0		
cellular component	0	0	0	0	0	0	0	0	0	
organism model	0	0	0	0	0	0	0	0	0	0

Table S3

Resource: CIDeR
Disease: Parkinson
Interactions: 3235

	Gene/Protein	tissue/cell line	process	phenotype	microRNA	environment	drug/chemical compound	disease	cellular component	organism model
Gene/Protein	1183									
tissue/cell line	27	0								
process	701	4	58							
phenotype	94	1	18	0						
microRNA	10	4	0	1	0					
environment	0	0	0	0	0	0				
drug/chemical compound	394	10	218	19	2	0	47			
disease	223	0	24	17	0	0	13	1		
cellular component	151	0	2	1	0	0	8	0	0	
organism model	2	1	1	0	0	0	0	0	0	0

Resource: CTD
Disease: Parkinson
Interactions: 47399

	genes/proteins	tissue/cell line	process	phenotype	microRNA	environment	drug/chemical compound	disease	cellular component	organism model
genes/proteins	0									
tissue/cell line	0	0								
process	0	0	0							
phenotype	0	0	0	0						
microRNA	0	0	0	0	0					
environment	0	0	0	0	0	0				
drug/chemical compound	0	0	0	0	0	0	0			
disease	42940	0	0	0	0	0	4459	0		
cellular component	0	0	0	0	0	0	0	0	0	
organism model	0	0	0	0	0	0	0	0	0	0

Resource:	KEGG
Disease:	Parkinson
Interactions:	11

	genes/proteins	tissue/cell line	process	phenotype	microRNA	environment	drug/chemical compound	disease	cellular component	organism model
genes/proteins	11									
tissue/cell line	0	0								
process	0	0	0							
phenotype	0	0	0	0						
microRNA	0	0	0	0	0					
environment	0	0	0	0	0	0				
drug/chemical compound	0	0	0	0	0	0	0			
disease	0	0	0	0	0	0	0	0		
cellular component	0	0	0	0	0	0	0	0	0	
organism model	0	0	0	0	0	0	0	0	0	0

Table S4

Resource: CIDeR
Disease: Amyotrophic Lateral Sclerosis
Interactions: 2414

	genes/proteins	tissue/cell line	process	phenotype	microRNA	environment	drug/chemical compound	disease	cellular component	organism model
genes/proteins	1023									
tissue/cell line	77	0								
process	369	0	2							
phenotype	242	10	11	0						
microRNA	3	0	1	0	0					
environment	7	0	3	1	0	0				
drug/chemical compound	214	3	60	30	0	2	7			
disease	150	0	8	18	4	0	20	0		
cellular component	138	0	1	6	0	0	3	1	0	
organism model	0	0	0	0	0	0	0	0	0	0

Resource: CTD
Disease: Amyotrophic Lateral Sclerosis
Interactions: 5405

	genes/proteins	tissue/cell line	process	phenotype	microRNA	environment	drug/chemical compound	disease	cellular component	organism model
genes/proteins	0									
tissue/cell line	0	0								
process	0	0	0							
phenotype	0	0	0	0						
microRNA	0	0	0	0	0					
environment	0	0	0	0	0	0				
drug/chemical compound	0	0	0	0	0	0	0			
disease	3143	0	0	0	0	0	2262	0		
cellular component	0	0	0	0	0	0	0	0	0	
organism model	0	0	0	0	0	0	0	0	0	0

Resource: KEGG
Disease: Amyotrophic Lateral Sclerosis
Interactions: 40

	genes/proteins	tissue/cell line	process	phenotype	microRNA	environment	drug/chemical compound	disease	cellular component	organism model
genes/proteins	27									
tissue/cell line	0	0								
process	0	0	0							
phenotype	0	0	0	0						
microRNA	0	0	0	0	0					
environment	0	0	0	0	0	0				
drug/chemical compound	13	0	0	0	0	0	0			
disease	0	0	0	0	0	0	0	0		
cellular component	0	0	0	0	0	0	0	0	0	
organism model	0	0	0	0	0	0	0	0	0	0
