# Supplementary figures and images for: CIDeR: multifactorial interaction networks in human diseases
Source: Genome Biol. 2012 Jul 18;13(7):R62. doi: 10.1186/gb-2012-13-7-r62 (PMC3491383; doi:10.1186/gb-2012-13-7-r62)

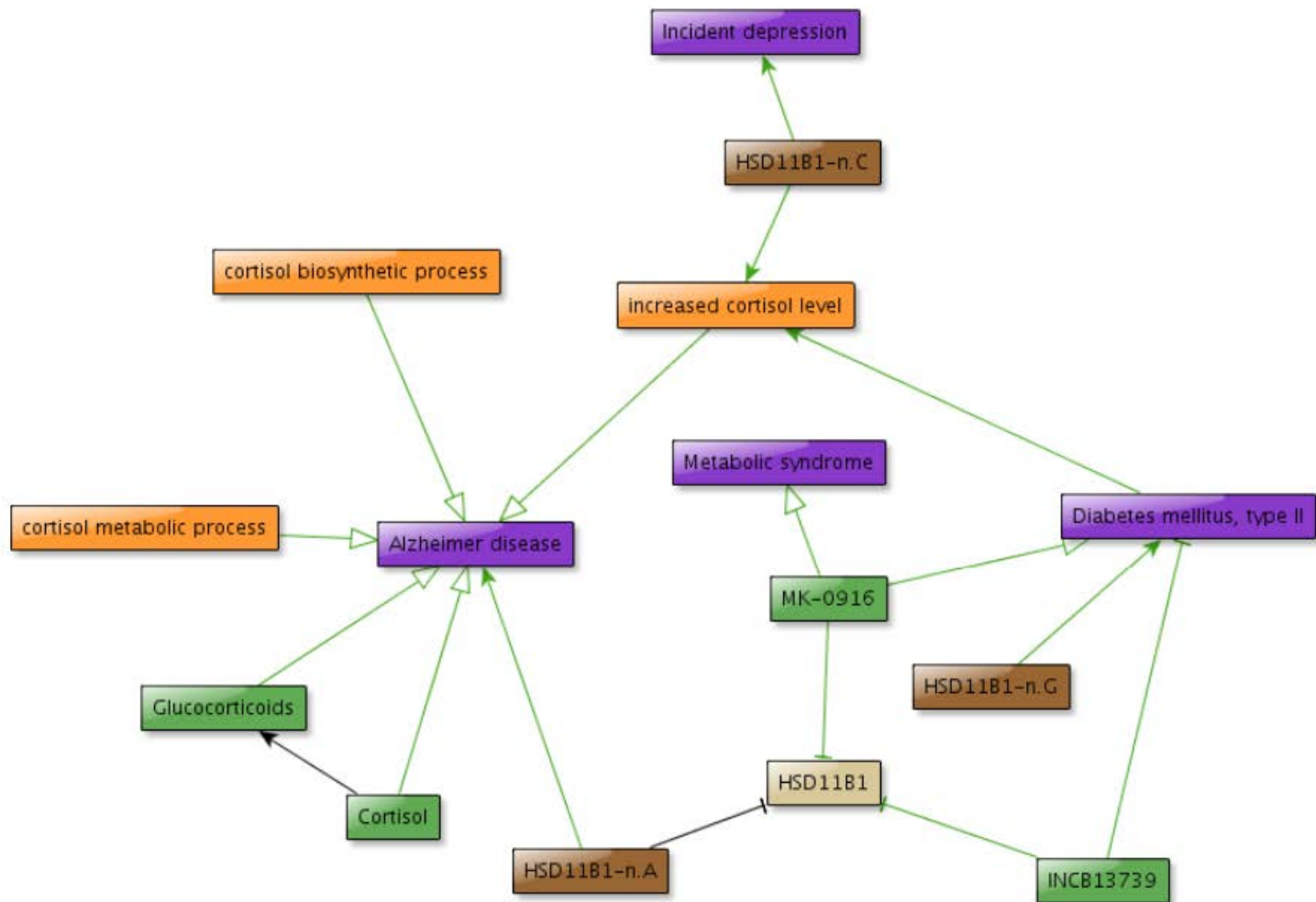

Supplement: Additional file 3 — Potential side effects of type 2 diabetes drugs on patients affected by Alzheimer's disease or depression. [file gb-2012-13-7-r62-S3.PDF]
